# Edible Cannabis and Pain, Sleep, and Mental Health Management in Older Adults

**DOI:** 10.1001/jamanetworkopen.2026.11718

**Published:** 2026-05-08

**Authors:** Rebecca K. Delaney, Melissa H. Watt, Madeline Stanger, Isabelle Hong, Nehal K. Bakshi, Angela Fagerlin, Angela Bryan

**Affiliations:** 1 University of Utah Intermountain Health Department of Population Health Sciences, Salt Lake City; 2 Department of Psychology and Neuroscience, University of Colorado Boulder, Boulder; 3 Now with Epic Systems, Madison, Wisconsin

## Abstract

**Importance:**

Older adults are the fastest-growing group of cannabis consumers in the US, seeking relief from age-related ailments (eg, pain or difficulty sleeping) and improved quality of life. However, little is known about the motivations and factors that influence their use of edible cannabis and product choice.

**Objectives:**

To explore the motivations of older adults in Colorado purchasing edible cannabis products to improve sleep, pain, or mental health concerns, and to understand how they perceive the benefits and drawbacks of different cannabinoid profiles: cannabidiol (CBD)–dominant, tetrahydrocannabinol (THC)–dominant, or a THC-CBD combination product.

**Design, Setting, and Participants:**

This community-based, qualitative study was study conducted in Colorado from November 2021 to November 2023 as part of a larger clinical trial. Individual interviews were audio-recorded, transcribed, and analyzed. Participants were a convenience sample of adults aged 60 years or older who were interested in using edible cannabis for sleep, pain, or mental health symptoms. Participants completed an in-person interview prior to purchasing 1 of 3 edible product types.

**Exposure:**

Use of edible cannabis products.

**Main Outcomes and Measures:**

The primary outcomes were self-reported motivations for cannabis use and perceived benefits and drawbacks of different cannabinoid profiles, assessed through semistructured interviews conducted before product purchase.

**Results:**

Among 169 participants (mean [SD] age, 70.8 [5.8] years, 89 female [54%]), 96 (57.5%) selected a THC and CBD combination product, 48 (28.7%) selected a CBD-dominant product, and 23 (13.8%) selected a THC-dominant product. Primary motivations for cannabis use included avoiding pharmaceuticals, exhausting other options, new or increasing problems, and evidence or claims of benefits. Evidence or claims of associated outcomes were seen as a benefit for all 3 product types. The most common drawback for THC and combination products was concern about getting high or being impaired, while limited efficacy was the most common drawback for CBD products. Combination products were perceived to offer dual benefits of THC-CBD, yet determining the ideal ratio was seen as a challenge.

**Conclusions and Relevance:**

In this qualitative study, older adults were motivated to use cannabis as an alternative approach to address health concerns. These findings highlight the importance of physician awareness of older adults’ motivations and concerns to support informed counseling and resources.

## Key Points

**Question:**

What motivates older adults in Colorado to purchase edible cannabis products for sleep, pain, or mental health symptoms?

**Findings:**

In this qualitative study of 169 older adults, most participants selected combination tetrahydrocannabinol (THC) and cannabidiol (CBD) (57.5%) or CBD-only (28.7%) products, rather than THC alone (13.8%). Primary motivations were avoiding pharmaceuticals and seeking symptom relief after other options were exhausted; concerns about associated psychoactive outcomes were common, particularly for THC-containing products.

**Meaning:**

The findings of this study suggest that older adults are increasingly turning to cannabis for symptom management; health care systems should consider motivations and concerns in physician guidance and resources.

## Teaser

This qualitative study explores why older adults in Colorado are motivated to purchase edible cannabis products for sleep, pain, or mental health concerns.

## Introduction

Cannabis use among older adults has been increasing since states began to legalize its use for medical and recreational purposes. Adults aged 60 years and older are increasingly exploring cannabis for both recreational purposes and the treatment of various health concerns.^1,2,3^ Pain, sleep problems, and negative mood or anxiety are the most common reasons older adults use cannabis for symptom relief.^4,5,6,7^ Despite patterns of increasing cannabis use among older adults, some do not disclose cannabis use with their medical practitioners,^6,8,9^ and practitioners themselves may be reluctant to initiate conversation or provide advice on cannabis use with patients due to a lack of knowledge.^10,11,12^ The lack of evidence on benefits and risks of cannabis use combined with the absence of clear medical guidance, makes it especially challenging for older adults to decide whether to use cannabis and which cannabinoid profile product to choose to meet their needs.

The legal market for cannabis offers products with varying cannabinoid profiles including those that primarily contain tetrahydrocannabinol (THC), cannabidiol (CBD), or are a combination of THC-CBD. THC is the cannabinoid associated with the euphoric or high feeling of cannabis,^13,14^ while CBD does not have these psychoactive effects.^15^ Early research on cannabis focused on THC, but CBD and combination products have gained popularity following more recent legalization.^16,17^ It is important to understand how older adults decide which cannabinoid profile to use and whether these choices reflect their motivations or concerns about potential harms and adverse effects.

In addition to smoking, edible products are a common method of cannabis use among older adults; however, less is known about older adults’ motivations and perceptions that inform edible cannabis product decisions.^6,18,19,20^ There is some research on older adults’ perceptions of positive (eg, alternative to prescription medications) and negative outcomes (eg, stigma) of cannabis use in general, but little is known about these perceptions specifically for edible products or across different cannabinoid profiles.^9,21^ Edible products pose distinct challenges for older adults, including delayed onset, dosing uncertainty, and risk of medication interactions, making it critical to understand decision-making around these products.^21^ Despite the growing use of edibles, little is known about how older adults choose between THC, CBD, or combination cannabis products or what motivations and concerns shape these decisions.

This study aims to explore the motivations and concerns of older adults who are deciding to purchase and use recreationally legal edible cannabis products to manage difficulties with sleep, pain, and mental health. Furthermore, this research aims to explore whether their motivations and concerns differ for THC-dominant products, CBD-dominant products, or combination THC-CBD products. Data from this study will guide the development of tools to provide evidence-based information that helps older adults align their choice with their values, preferences, and concerns.

## Methods

This qualitative study was conducted from November 2021 to November 2023 as part of a large cohort examining the associated outcomes of edible cannabis products on physical functioning, cognitive functioning, and well-being among older adults (NCT05188404). The study was approved by the institutional review boards at the University of Colorado at Boulder and the University of Utah. Written informed consent was obtained from participants, including permission for interviews to be audio-recorded and data to be published. This study followed the Standards for Reporting Qualitative Research (SRQR) reporting.^22^

Individuals were eligible if they were aged 60 years or older, not currently using cannabis more than 7 times per month, and interested in using cannabis for at least 1 of the following concerns: pain, sleep problems, depression, and/or anxiety. Demographic information was collected to characterize the study sample and provide context for interpreting participants’ perspectives. Participants were primarily recruited through direct mailings to older adults in zip codes within a 30-mile radius of Boulder, Colorado, including the Denver metropolitan area. Addresses were purchased from a marketing firm and recruitment was supplemented by flyers and community outreach events.

Enrolled participants self-reported demographic information in a survey and completed a brief semistructured interview that included questions about why they were interested in trying cannabis and their perceptions about different types of edible cannabis products (THC, CBD, or combination) (eMethods in Supplement 1). Participants selected from multiple options to self-report race and ethnicity, including an option for preferring not to answer the question. Interviews were conducted primarily by 1 trained research staff member (M.S.) who received formal training in qualitative interviewing from senior qualitative investigators (M.H.W. and R.K.D.), including instruction on the study-specific interview guide. A second trained team member conducted a limited number of interviews as needed. Following the interview, participants were shown an infographic with brief information about the 3 different types of edible cannabis products and asked to choose which product type they intended to purchase from a local dispensary. The interviews were audio-recorded and transcribed verbatim.

### Data Analysis

We conducted directed qualitative content analysis using a structured codebook approach.^23^ Initial coding domains were informed by the interview guide (eg, motivations for cannabis use, perceived benefits, and perceived drawbacks), and themes within domains were identified inductively through team-based analysis. Members of the analytic team (R.K.D., M.H.W., I.H., and N.K.B.) reviewed and discussed the first 20 transcripts to create an initial codebook that had domains as parent codes (eg, motivations for using cannabis and concerns about cannabis) and emerging themes as child codes. We used Nvivo software version 14 (Lumivero) to facilitate organization and coding of the transcripts. The first half of the transcripts were double coded, and the 2 coders (I.H. and N.K.B.) met to reach consensus on coding. Regular team discussions were held to review coding decisions and refine thematic interpretations. If coders had challenges reaching consensus, or if they agreed that the content necessitated a new code, then this was discussed with the lead qualitative researchers (M.H.W. and R.K.D.). The larger study aimed to enroll approximately 300 participants to support quantitative analyses. Interviews were conducted at baseline alongside surveys and additional assessments. Ongoing qualitative analysis indicated that thematic saturation had been achieved at approximately the midpoint of enrollment. To reduce participant burden while maintaining analytic rigor, the interviews were discontinued at baseline, and recruitment continued for the quantitative component of the larger study. The final qualitative sample provided sufficient depth and variability to support stable thematic development.

Following coding, we ran queries to examine code output, confirm the distinction among codes, and merge codes that had shared meaning. We generated matrix outputs to examine the application of codes across categories by product choice. Given the nature of qualitative interviews and analysis, some participant responses were coded with multiple themes in a single domain (eg, if their response reflected more than one idea), and some participants’ responses were not coded with any theme in a domain (eg, if participant did not answer the question). As a result, the total number of participants represented in each theme varies, and percentages do not add up to 100%. In reporting our data, we prioritized the most prominent themes that emerged from the data, as these represented the most salient and recurrent patterns in participants’ experiences and perspectives.

## Results

Among 169 participants, the mean (SD) age was 70.8 (5.8) years (age range, 60-85 years) and 89 (54%) identified as female (Table 1). Overall, there were 3 Asian (2%), 4 biracial or multiracial (2%), and 154 White (93%) participants. Eighty participants reported using cannabis an average of 1.29 times per week. Interviews lasted mean (SD) approximately 11.2 (1.3) minutes (range 9.4-14.3 minutes) on average. All participants agreed to audio-recordings, and no repeat interviews were conducted.

**Table 1.** Demographic Information of Participants

Characteristic	Participants No. (%) (N = 169)
Age, mean (SD), y^a^	70.8 (5.8)
Gender^a^	
Female	89 (54)
Male	77 (46)
Marital status^b^	
Married	103 (62)
In a relationship (not married)	8 (5)
Living with partner (not married)	4 (2)
Divorced	18 (11)
Separated	1 (1)
Single	22 (13)
Widowed	11 (7)
Education level^a^	
High school degree or General Education Development test	2 (1)
Some college	19 (11)
Associate’s degree or Technical Certification degree	6 (4)
Bachelor’s degree	41 (25)
Master’s degree	73 (44)
Doctoral degree	25 (15)
Race and ethnicity^a^	
American Indian or Alaska Native	1 (1)
Asian or Asian American	3 (2)
Biracial or multiracial	4 (2)
White	154 (93)
Prefer not to answer	4 (2)
Hispanic^a^	
Yes	9 (5)
No	149 (90)
Prefer not to answer	8 (5)

^a^ Missing data from 3 participants.

^b^ Missing data from 2 participants.

**Figure 1.**
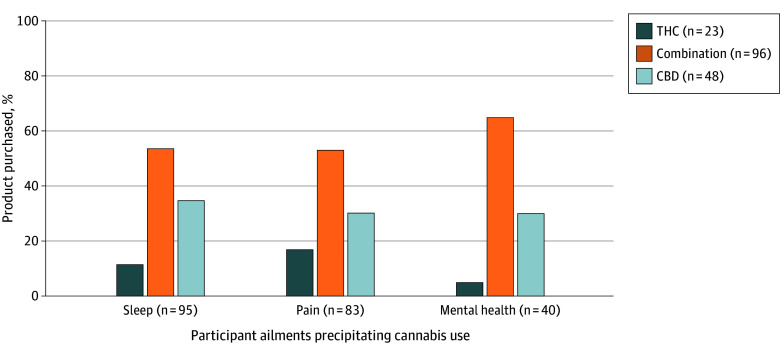
Bar Chart Illustrating Ailments Precipitating Cannabis Usage Figure shows ailments for which participants wanted to use cannabis by cannabis product purchased (n = 167). CBD indicates cannabidiol; and THC, tetrahydrocannabinol.

When asked about the symptoms or health issues that led them to try a cannabis product, 95 (56.8%) said they wanted to use cannabis for sleep, 84 (49.7%) for pain, and 42 (24.9%) for mental health (Figure 1). Most participants reported that they planned to purchase a combination product (96 participants [57.5%]), followed by CBD (48 participants [28.7%]) and THC (23 participants [13.8%]). Across all symptom categories, pain, sleep, and mental health, combination CBD-THC products were the most selected. Among the remaining options, CBD-dominant products were preferred over THC-dominant ones.

### Motivations for Using Cannabis

**Figure 2.**
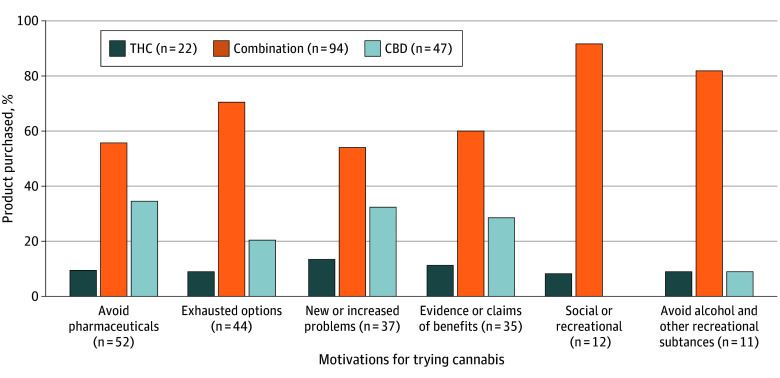
Bar Chart Illustrating Motivations for Trying Cannabis Figure shows motivations for trying cannabis, by intended choice of cannabis product (n = 165). CBD indicates cannabidiol; and THC, tetrahydrocannabinol.

Six themes were identified related to motivations for trying edible cannabis products. Themes and exemplary quotations are included in Table 2. Many participants described a reluctance in using traditional pharmaceutical treatments. Notably, they had concerns about adverse effects, long-term health risks, or dependency associated with pharmaceutical medications and viewed cannabis as a safer alternative. Participants also reported that they had exhausted all pharmacologic and nonpharmacologic options (eg, therapy, acupuncture, or massage) for symptom management so they desired to try cannabis as a last resort. Some participants who were experiencing substantial physical and mental health burdens sought to use cannabis to address their new or escalating symptoms related to pain, sleep disturbances, or mood changes. Participants also heard about benefits of cannabis use through personal networks, medical talks, and media sources that further sparked motivation for cannabis use. A smaller number of participants spoke about wanting to use cannabis for recreational use such as for getting high or to improve social gatherings with friends and activities. Some wanted to substitute cannabis for other recreational substances, such as alcohol, to enhance their mood or avoid negative physiological outcomes of other substances. Across all motivations, older adults were most likely to choose a combination cannabis product, but distributions of motivations differed by the product participants intended to purchase (Figure 2).

**Table 2.** Exemplary Quotations for Different Motivations for Trying Cannabis

Motivations	No. of participants (N = 169)	Exemplary quotations (decade of life, product they intended to purchase)
Seeking alternatives to pharmaceutical medications	54	“I guess because I don’t want to go on psychiatric medications. And I know that other things that have helped me with sleep before have a correlation with cognitive decline such as Benadryl. There’s some research that shows that that really can affect your brain and your cognitive functioning. So I think cannabis is probably a better choice.” (70s, Combination product)
		“I worry about the side effects of the NSAID meds, the Aleve, Excedrin, aspirin, ibuprofen. They all really do help my arthritis when I take it, but I’ve also had friends that have gotten bleeding ulcers from taking those meds too much. So that’s made me very worried about taking them too often.” (70s, Combination product)
Exhausted other options	46	“Most everything else is either ineffective or doesn’t clear your system. I’m a little concerned about alcohol as a sleep aid because it’s toxic and affects your kidneys and liver and all kinds of other things. And I found that melatonin isn’t that effective. I don’t really want to do prescription, over-the-counter drugs. I’ve done those in the past and they just make you groggy the next day…” (60s, Combination product)
		“Only because I’ve gone through so much to try to have the pain taken away. Like with steroid injections, massages, Tramadol, therapy, yoga…” (60s, Combination product)
		“I’ve just tried so many things to deal with anxiety that this is like the last frontier. I like to know if something really does work that doesn’t leave you with a hangover or foggy or something that just calms me.” (60s, CBD product)
Worsening or new age-related symptoms	40	“As I am aging I have some joint pains that I would like to get rid of. I’m very active. I’d rather not have those. They are kind of adding up. I’ve had lower back problems for many, many years…I added some hip problems, some hip pain problems and lately a little shoulder problem.” (60s, CBD product)
		“Especially in the last ten years or so I’ve become much more achy and much more depressive in the mornings. My sleep quality is lower than it used to be. So, that’s the motivation.” (60s, Combination product)
Influence of evidence or social claims of benefit	36	“Because I’ve read about it and I have friends who are on medical cannabis who are getting relief, getting help with sleep and some relief from pain.” (70s, Combination Product)
		“I’ve attended various talks on cannabis use from medical doctors and I’ve read and heard that cannabis, or CBD anyway, can help with sleep.” (60s, CBD product)
Interest in social or recreational use	12	“I wouldn’t mind getting high, see what that’s like. I mean I remember that from the old days. Just recreational would be nice.” (70s, Combination product)
		“Social, just social with friends, yeah, have fun. Enjoy music more…just getting together with friends, going to see music, hiking, I really enjoy it when I’m hiking, being out in the woods.” (60s, Combination product)
Substance substitution	11	“Well, I’m looking for something that’s not alcohol. I’d rather not drink alcohol at all, and I think cannabis is a better choice just as a mood lifter.” (60s, Combination product)
		“Most everything else is either ineffective or doesn’t clear your system. I’m a little concerned about alcohol as a sleep aid because it’s toxic and affects your kidneys and liver and all kinds of things.” (60s, Combination product)

Abbreviations: CBD, cannabidiol; NSAID, nonsteroidal anti-inflammatory drugs.

### Perceived Benefits and Drawbacks by Product Type

Multiple product-specific themes related to perceived benefits and drawbacks were identified. Table 3 summarizes the most prominent themes for each product type including exemplar quotations. For THC products, 3 benefit themes and 6 drawback themes emerged. Perceived benefits of THC products centered on psychoactive effects and therapeutic impact. Participants described perceptions that THC-dominant products can improve relaxation, mood, and experiences through the psychoactive effects of getting high. Anecdotal or clinical claims supporting use were also commonly referenced as a benefit. Drawbacks focused primarily on impairment and unwanted psychoactive effects or adverse effects (eg, increased anxiety), including concerns about cognitive impact, dependency, and reduced motivation. Participants overall were concerned about how THC-dominant products would interfere with daily functioning.

**Table 3.** Product-Specific Themes Related to Perceived Benefits and Drawbacks (N = 169)^a^

Type of product and theme	Participants, No.	Exemplary quotation (decade of life, product they intended to purchase)
THC product		
Perceived benefits		
Psychoactive effects	66	“I do think they are relaxing and sort of change your perspective on life in that moment. I think they have an ability to help you sort think differently about things.” (60s, Combination product)
Anecdotal evidence or claims of impact	60	“For people that are terminally depressed. I’ve been around a lot of posttraumatic stress individuals, like a lot. They are used in conjunction with therapy and then finding other ways to operate so they can get out of the hole far enough to operate.” (80s, CBD product)
Substitute for other recreational substances	7	“Because I don’t think it’s dangerous and I do enjoy a little light pick me up from alcohol so that might be kind of nice, as a substitute for alcohol.” (60s, Combination product)
Perceived drawbacks		
Concerns about functional impairment	60	“My experience is sometimes it has a lasting effect in the morning and it also can be detrimental if you want to do anything, you know, like driving or anything else.” (60s, CBD product)
Unwanted psychoactive effects	55	“My past experience has been that (being high) sometimes makes me physically uncomfortable.” (70s, Combination product)
Concerns about adverse effects	31	“I know that THC is a product in the past that has really elevated my paranoid and anxiousness and appetite like I said.” (60s, CBD product)
Potential cognitive harm	20	“Curious about memory and cognitive function, either while using or long term. Is it doing something to reduce cognition?” (60s, Combination product)
Increased risk of dependency	23	“Dependency could develop, psychological dependency could develop.” (60s, Combination product)
Lack of energy or motivation	15	“I’ve known people that have smoked a lot of pot and, it’s stereotypical. They get lethargic, they slow down, they don’t get things done, and they forget things.” (70s, Combination product)
CBD product		
Perceived benefits		
Anecdotal evidence or claims of impact	114	“I’ve heard, you know, I know people who use CBD who swear by it to help them through whatever their issues are for the day.” (60s, Combination product)
Ability to avoid or minimize functional impairment	22	“Not getting high and being able to function in society, normal functioning and being able to be out and about driving and doing activities cognitively. I guess that’s a nice advantage to that.” (70s, Combination product)
Perceived drawbacks		
Uncertainty about efficacy	71	“The only drawback is if it’s ineffective, and it’s just kind of psychosomatic or whatever, like a placebo.” (60s, CBD product)
Cost burden	16	“The cost of it, I didn’t want to buy a $60 bottle of something only to have it not work.” (60s, CBD product)
No psychoactive effects	10	“The only drawback I would see would be just not being able to experience – again, let’s call it a high, but I prefer to use a different word.” (60s, THC product)
Combination product		
Perceived benefits		
Complementary effects of 2 products	70	“Because I think if the benefits are in the CBD and not in the THC or the THC and not in the CBD, if it’s a combination at least I should be able to get the benefits from something.” (60s, Combination product)
Anecdotal evidence or claims of impact	31	“From a physiological perspective what I have heard and read is that it can be more medicinally valuable to use the full spectrum of cannabinoids and whatever else.” (60s, CBD product)
Dosing flexibility	12	“I think anything in combination can just be more specific to the person. So it seems to me that prescribed drugs are kind of a blanket pill for everybody, so I like the idea of it being essentially tailor-made.” (60s, CBD product)
Perceived drawbacks		
Unwanted psychoactive effects	25	“I’m not looking to get high from the product, so I would say if it had too much THC in it then I would have to make sure I wasn’t going to drive, wasn’t going to use a power tool, or something.” (60s, CBD product)
Difficulty with tailoring products and ratios	19	“Well, it would be a little more difficult to figure out which was responsible for whatever I was experiencing. There would be less clarity in what I was doing.” (70s, THC product)
Uncertainty about efficacy	14	“It might not work. You know, a singular product might work better; I have no idea.” (60s, Combination product)
Concerns about functional impairment	10	“How high will I get? Will it affect my activities of daily living? Are there things I’m going to have to really pay attention to with the THC in there?” (60s, Combination product)

Abbreviations: CBD, cannabidiol; THC, tetrahydrocannabinol.

^a^ Many individuals said that they did not know of any benefits or drawbacks across the product types.

For CBD products, 2 benefit themes and 3 drawback themes were observed. For CBD-dominant products, anticipated therapeutic benefit was the most frequently described advantage, often informed by anecdotal evidence from peer or media accounts, which were similarly mentioned as a theme for THC-dominant product benefits. Participants also valued the absence of impairment. Although participants frequently referenced claims of therapeutic benefit, limited efficacy emerged as a dominant drawback, highlighting ambivalence about the reliability or magnitude of these effects. Concerns about the cost of products and lack of psychoactive effects also emerged as drawbacks for CBD.

For combination products, 3 benefit themes and 4 drawback themes were identified. Combination products were viewed favorably for offering complementary and synergistic benefits of both THC and CBD products, including the ability to adjust the ratios and tailor to their needs. Like THC-dominant and CBD-dominant products, participants reported that they had heard evidence of combination products having therapeutic effects. However, participants expressed concerns about being able to choose a ratio of the 2 product types that balanced the benefits and drawbacks of both product types. Similar concerns emerged about impairment, uncertainty about dosing, and potential ineffectiveness that were discussed about THC-dominant and CBD-dominant products. Overall, participants appeared to evaluate product types by weighing anticipated therapeutic benefit against concerns about impairment, efficacy, and functional impact.

## Discussion

As cannabis legalization becomes more widespread, older adults are increasingly turning to it not just for recreational use, but to manage symptoms associated with aging, including pain, sleep disturbances, and mental health concerns. In the absence of medical consultation, they face numerous decisions without clear information about what product can best address their needs. This qualitative study explored older adults’ motivations to try edible cannabis and their perceptions about products with different cannabinoid profiles (THC- dominant, CBD-dominant, and combination). Combination products (THC and CBD) were selected the most to address the 3 different ailments (sleep, pain, and mental health) with some variability for selection of cannabinoid-dominant (THC or CBD) products to address different ailments. Participants shared that they were motivated to try edible cannabis because they wanted to avoid pharmaceuticals, had exhausted other options, were experiencing new or increasing health problems, and had heard about benefits of cannabis. These motivations align with prior research showing that older adults are increasingly curious about and open to using cannabis to address the challenges of aging, reflecting a broader trend toward seeking alternatives to pharmaceuticals.^4,9^

Older adults were motivated to use cannabis as an alternative to traditional pharmaceuticals due to concerns about adverse effects and ineffectiveness of medications they have tried previously. Because health care practitioners generally feel unequipped to offer guidance on cannabis use,^10,11,12^ older adults rely on advertising, personal experience, and/or anecdotal claims of benefits to guide their decision to use cannabis products for treatment. As they weigh decisions about the type of cannabinoid that would best meet their needs, they must consider what they perceive as the strengths and drawbacks of each product type. Because older adults tend to focus on positive, emotional aspects from word-of-mouth or personal use,^24,25,26^ they may place greater weight on the strengths of cannabis over drawbacks or effectiveness. The consequences of this decision-making process are unclear if the product does not meet their expectations of relief. For example, participants may feel they have wasted time and money on a product that failed to provide relief. Future analyses with this cohort will explore experiences with edible cannabis products longitudinally to examine whether participants’ assessments of the potential benefits and drawbacks of a cannabis product change after using the product.

Participants expressed nuanced views of the potential benefits and drawbacks of different cannabinoid profiles (eg, CBD-dominant, THC-dominant, and combination of THC and CBD). Across all 3 product types a primary motivation for cannabis use was the belief, often informed by product marketing or anecdotal evidence, that cannabis could manage symptoms such as sleep difficulties, pain, anxiety, and depression. Although evidence of the efficacy of different cannabinoid profiles is limited, this perception reflects prior research showing that older adults are primarily using cannabis for these concerns.^4,5,6,7^ Our findings suggest that advertising and word-of-mouth experiences could be shaping older adults’ beliefs about which cannabinoids address particular symptoms. Among those selecting THC-dominant products, pain was the most frequently targeted symptom. THC-CBD combination products were most chosen overall, possibly suggesting a desire to address multiple symptoms simultaneously. These patterns highlight how older adults are making cannabis-associated decisions without consistent medical guidance. More research and clinical support are needed to help them make informed choices aligned with their health needs.

Considerations for benefits and drawbacks of each cannabinoid profile that align with older adults’ preferences add further complexity to making decisions. Different perceptions emerged for concerns by cannabinoid profile type: concerns about getting high or impaired were largely discussed for THC-dominant products and combination products, whereas CBD-dominant products were considered less effective. Combination products were perceived to be a goldilocks option with shared benefits of THC and CBD. However, participants also expressed concerns about getting an ideal ratio of THC and CBD to address their unique symptoms. Other research has found that while some older adults would vary levels of CBD and THC to meet their needs, it was difficult to do at times with limited information on product labels and suggested the need for informational pamphlets to clarify dosages.^21^

### Limitations

Despite providing new insight into older adults’ motivations and perceptions regarding decisions between different cannabinoid profiles, this study has limitations. First, the sample was highly educated, had a high proportion of non-Hispanic White individuals, recruited from a limited catchment area within Colorado, and from a state where recreational cannabis is legalized. While the sample demographics broadly reflect the study catchment area, these findings may be less generalizable to more diverse populations and other states where recreational cannabis remains illegal. Second, given the focus was on edible cannabis products, perceptions could be different for other types of cannabis administration (eg, inhalants or topical products). Third, there was heterogeneity in the participants’ recent cannabis use, which could have influenced participants’ expectations about different products, worry or anxiety about product selection or dosing, and perceptions of physiological effects. Future research could examine how varying levels of prior cannabis use, including frequency of behaviors and product purchasing history impact product preferences, subsequent experiences with products, and symptom management outcomes. Fourth, the study included participants who were specifically interested in cannabis use to address pain, sleep, or mental health concerns, therefore missing viewpoints from individuals who wanted to use cannabis for other ailments or for more general wellness.

## Conclusions

In this qualitative study of older adults, participants described using edible cannabis as an alternative approach to address health concerns and expressed varied views about the benefits and drawbacks of different cannabinoid profiles. The findings highlight the need for stronger clinical evidence, patient-centered guidance, and accessible education to help older adults confidently select edible cannabis products to address pain, sleep, or mental health concerns. Older adults sought alternatives to the pharmaceuticals they were currently using, either because of a lack of efficacy or undesirable adverse effects. With the most common product profile selected being a combination of CBD and THC, expanding research to identify the potential benefits and harms of this treatment option may help inform clinical guidance. Future efforts should focus on equipping practitioners with practical tools and creating accessible patient resources to ensure older adults can make informed choices about edible cannabis products as part of their care.

## Notes

Supplement 1. eMethods. Semi-Structured Interview Guide

Supplement 2. Data Sharing Statement
