# Supplementary material for: Edible Cannabis and Pain, Sleep, and Mental Health Management in Older Adults
Source: JAMA Netw Open. 2026 May 8;9(5):e2611718. doi: 10.1001/jamanetworkopen.2026.11718 (PMC13156787; doi:10.1001/jamanetworkopen.2026.11718)
Supplement: Supplement 1. — eMethods. Semi-Structured Interview Guide [file jamanetwopen-e2611718-s001.pdf]

Supplemental Online Content

Delaney RK, Watt MH, Stanger M, et al. Edible cannabis and pain, sleep, and mental health management in older adults. *JAMA Netw Open.* 2026;9(5):e2611718. doi:10.1001/jamanetworkopen.2026.11718

eMethods. Semi-Structured Interview Guide

This supplemental material has been provided by the authors to give readers additional information about their work.

© 2026 Delaney RK et al. *JAMA Network Open*.

eMethods. Semi-Structured Interview Guide

This semi-structured interview guide was used in individual in-person interviews conducted with adults aged 60 years or older prior to purchasing an edible cannabis product. Interviews were conducted by trained research staff and were audio-recorded and transcribed verbatim. The guide included open-ended questions and optional probes to explore motivations for cannabis use and perceptions of different cannabinoid profiles (cannabidiol [CBD]–dominant, tetrahydrocannabinol [THC]–dominant, and combination THC/CBD products).

In this part of the visit, I'm going to be asking you some questions about your motivations to use cannabis and your thoughts on different types of cannabis products. Please feel comfortable to speak freely. There are no right or wrong answers. And if you don't know how to answer a question, or you would prefer to skip a question, that's ok. Are you ready to start?

1. When you think about using cannabis, how are you hoping it will help you? (PROBE: anything else)

2. When you think about using cannabis, what are you concerned about? (PROBE: anything else)

3. People choose to try cannabis for many different reasons. What's your main motivation for trying cannabis at this point in your life? (PROBE: why cannabis, instead of other products or strategies?)

4. Typically cannabis products come in 3 different forms: They're either mostly THC, mostly CBD, or they're a combination of both THC and CBD. I'd like to get your thoughts on the three different types of cannabis products, and whether you think the product is a good fit for your needs.

   THC: First, let's talk about THC products.

   a. What's your opinion about cannabis products that are mostly/all THC? **[If participant says they don't know anything about THC, remind them of the survey definition: THC stands for tetrahydrocannabinol and can produce the "high" someone feels from using cannabis]**
      - What do you see are benefits to this product?
      - What do you see as drawbacks to this product?
   b. Do you think it's a good fit for your needs?
      - Why / why not?

   CBD: Now, let's talk about CBD products.

   a. What's your opinion about cannabis products that are mostly/all CBD? **[If participant says they don't know anything about CBD, remind them of the survey definition: CBD stands for cannabidiol and does not produce a "high."]**
      - What do you see are benefits to this product?
      - What do you see as drawbacks to this product?
   b. Do you think it's a good fit for your needs?
      - Why / why not?

© 2026 Delaney RK et al. *JAMA Network Open*.

THC + CBD: Thank you for your thoughts on THC and CBD products. As you may know, you can also get products that combine both THC and CBD.

a. What's your opinion about products that are a combination of both THC and CBD?
   - What do you see are benefits to this product?
   - What do you see as drawbacks to this product?

a. Do you think it's a good fit for your needs?
   - Why / why not?

5. I really appreciate your thoughts about the three different types of cannabis products. As you can imagine, people can find it difficult to choose the cannabis product that is best for them. What information do you think people need, so that they can choose the right product for their needs?

6. What do you think is the best way for people to learn about the different cannabis products?

7. Have you ever spoken to a health care provider about cannabis use?

   IF YES:
   - What was helpful when you spoke to your provider about cannabis use?
   - What was challenging or difficult when you spoke to your provider about cannabis use?

   IF NO:
   - Why have you not spoken to a health care provider about cannabis?
   - Would you find it valuable to talk to a provider about cannabis use in the future? Why or why not?
   - What might help you have these conversations in the future?

8. I've finished asking you my questions about selecting cannabis products. Is there anything else you'd like to tell me about this topic?

© 2026 Delaney RK et al. *JAMA Network Open*.
