# Supplementary material for: Edible Cannabis and Pain, Sleep, and Mental Health Management in Older Adults
Source: JAMA Netw Open. 2026 May 8;9(5):e2611718. doi: 10.1001/jamanetworkopen.2026.11718 (PMC13156787; doi:10.1001/jamanetworkopen.2026.11718)
Supplement: Supplement 2. — Data Sharing Statement [file jamanetwopen-e2611718-s002.pdf]

Data Sharing Statement

Delaney. Edible Cannabis and Pain, Sleep, and Mental Health Management in Older Adults. *JAMA Netw Open*. Published May 08, 2026. doi:10.1001/jamanetworkopen.2026.11718

Data
Data available: Yes
Data types: Deidentified participant data
How to access data: De-identified qualitative data (coded interview memos and the final codebook) will be made available upon reasonable request to the corresponding author after publication (rebecca.delaney@hsc.utah.edu). Audio recordings and any materials containing personally identifiable information will not be shared to protect participant confidentiality.
When available: With publication

Supporting Documents
Document types: None

Additional Information
Who can access the data: Access will be granted to researchers for research purposes under a data use agreement and with documentation of IRB approval or exemption as applicable.
Types of analyses: Access will be granted to researchers for research purposes under a data use agreement and with documentation of IRB approval or exemption as applicable.
Mechanisms of data availability: Access will be granted to researchers for research purposes under a data use agreement and with documentation of IRB approval or exemption as applicable.
